# APOBEC3G & HTLV-1: Inhibition without deamination

**DOI:** 10.1186/1742-4690-2-37

**Published:** 2005-05-29

**Authors:** Klaus Strebel

**Affiliations:** 1 Laboratory of Molecular Microbiology, Viral Biochemistry Section; National Institute of Allergy and Infectious Diseases, NIH; Building 4, Room 310; 4 Center Drive, MSC 0460; Bethesda, MD 20892-0460, USA

## Abstract

APOBEC3G is a cellular cytidine deaminase that was recently identified as the Vif-sensitive antiviral host factor responsible for the restriction of *vif*-defective HIV-1 in primary human cells and certain non-permissive T cell lines. Inhibition of HIV-1 replication is thought to be the result of APOBEC3G-induced hypermutation of the viral genome that occurs early during reverse transcription. Against this backdrop is a new report from the Uchiyama laboratory that proposes deaminase-independent restriction of HTLV-1 by APOBEC3G (Sasada et al. Retrovirology 2005, **2**:32). These findings combined with recent reports of deaminase-independent inhibition of Hepatitis B virus as well as HIV-1 suggest that cytidine deaminase activity and antiviral activity may be separable functional properties of APOBEC3G.

The identification of APOBEC3G (APO3G) in 2002 as the long elusive cellular target of the HIV viral infectivity factor (Vif) [1] has triggered an outburst of research activity that has produced in a short period of time a rather comprehensive working model for APO3G function (Fig. 1). Although the details of this model are changing almost daily, it is generally believed that APO3G does not interfere with virus production from infected cells but acts at a post-entry level to prevent the productive infection of new target cells. This model is consistent with more than a decade worth of biological and genetic data demonstrating that the Vif-sensitive host factor inhibits HIV-1 infectivity when expressed in the virus-producing cell but does not inhibit infection by HIV-1 when expressed in the target cell (for review see [2]). It is clear that the antiviral activity of APO3G requires its presence in progeny virions where it can cause hypermutation of the viral genome during the reverse transcription step early after infection. Hypermutation of the viral genome at that stage of the viral replication cycle is thought to either result in mutational inactivation of the viral genome – ensuing in the production of defective virions in the next replication cycle – or to trigger degradation of the viral genome in the target cell by activating a host DNA repair mechanism thus resulting in abortive infection (reviewed in [3]).

**Figure 1:**
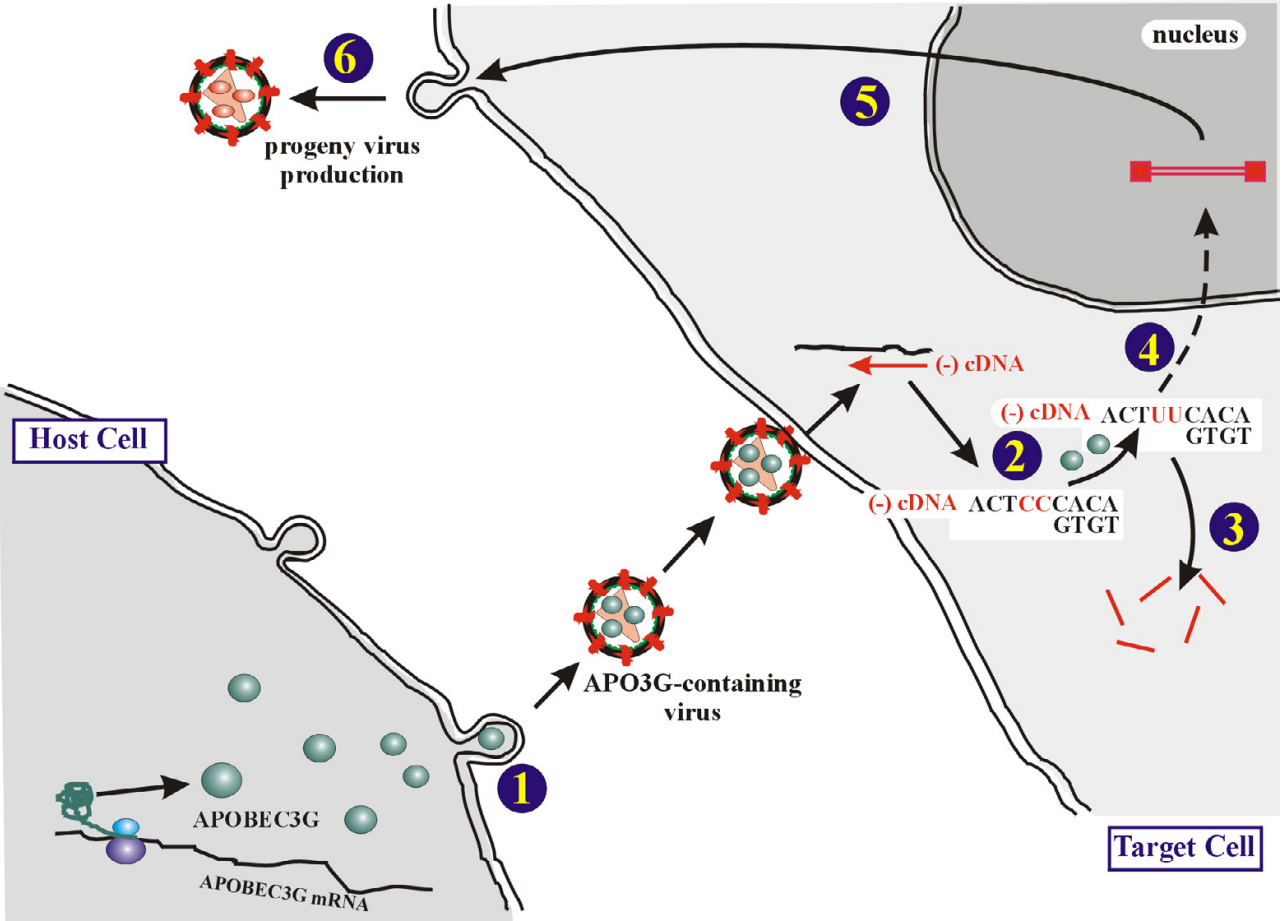
Inhibition of virus infectivity by APO3G. Cells restrictive for the replication of Vif-defective HIV express the cytidine deaminase APOBEC3G (APO3G). In the absence of Vif, APO3G is packaged into virus particles (**1**). Such virions are capable of penetrating a target cell and initiate minus-strand cDNA synthesis ((-)-cDNA). However, APO3G causes hypermutation of the viral (-)-cDNA resulting in the conversion of deoxycytidine to deoxyuridine (**2**). Deoxyuridine residues in the viral cDNA can be targeted by uracil-DNA glycosylase, which could lead to endonucleolytic cleavage by endonucleases present in the target cell (**3**). Alternatively, hypermutated cDNA enters the nucleus (**4**) and integrates into the host genome but results in the production of defective or aberrant viral proteins (**5**). This can lead to an impairment of virus assembly or result in the assembly of non-infectious viruses (**6**).

Primate lentiviruses such as HIV and SIV have adapted to APO3G with the help of the virus-encoded *vif *gene whose function is to prevent the packaging of APO3G into virus particles (reviewed in [3]). However, APO3G can target other viruses that do not encode a *vif *gene or a Vif-like activity. Indeed, APO3G was shown to inhibit the replication of Hepatis B virus (HBV) [4] and was found to affect the retrotransposition of endogenous retroviruses alike [5]. Inhibition of retrotransposition was correlated with G-to-A hypermutation of the endogenous retroviral genomes [5] and APO3G-induced hypermutation of HBV genomes was observed at least in one cell type [6]. However, APO3G-induced hypermutation of the HBV genome seemed to be rare and inhibition of HBV by APO3G appeared to be primarily due to the suppression of viral DNA synthesis through a deamination-independent mechanism [4]. The precise mechanism of such deamination-independent suppression of HBV by APO3G remains elusive. Also, the functional relevance of the APO3G-based restriction of HBV replication *in vivo *remains to be determined given the fact that expression of APO3G in human hepatocytes – the primary target for HBV – is very low.

Against this backdrop appeared a study by the Uchiyama laboratory investigating the potential antiviral activity of APO3G towards HTLV-1 (Sasada et al. Retrovirology 2005, **2**:32). HTLV-1 differs from HIV-1 in that it produces only very low levels of cell-free infectious virions suggesting a mode of virus transmission that is dependent on close cell-to-cell contacts [7,8]. Interestingly, the genetic diversity of HTLV-1 is much lower than that of HIV-1 even though both viruses target primarily APO3G-expressing cells and despite the fact that HTLV-1 does not appear to encode a gene with Vif-like function. Similar resistance to APO3G was observed for MuLV, which replicates in APO3G-expressing murine cells without accumulation of hypermutations despite the fact that murine APO3G is packaged into MuLV virions [9]. These results suggest that packaging of APO3G into viral particles per se may not be sufficient to inhibit viral infectivity. Rather, it seems that APO3G has to be specifically packaged into the viral core in association with the viral RNA to exert its inhibitory activity [10]. In the new study, Sasada et al report that APO3G is efficiently packaged into HTLV-1 particles. This is true for endogenous and exogenously expressed APO3G alike. Interestingly, and consistent with the MuLV model, packaging of APO3G into HTLV-1 did not result in a significant accumulation of APO3G-induced hypermutations. In contrast to MuLV, however, Sasada et al note a profound effect of APO3G on the infectivity of HTLV-1 particles, which was reduced to almost background levels. Similar findings were recently reported by Navarro et al although the effects of APO3G on HTLV-1 infectivity in that study were found to be modest when compared to HIV-1 [11]. Surprisingly, Sasada et al found that variants carrying mutations in either the first or the second zinc finger domain of APO3G were capable of inhibiting HTLV-1 with similar efficiency than the wild type protein. This is in contrast to their previous finding that APO3G enzymatic activity was essential for anti-HIV-1 activity [12]. On the other hand, Newman et al recently demonstrated anti-HIV activity for deaminase-defective APO3G variants similar to the ones used in the current study [13]. Thus, while details have yet to be sorted out, there is an emerging picture of a multifunctional host factor that can exert antiviral activity by way of its inherent deaminase activity or through a deaminase-independent mechanism. One possible deaminase-independent mode of action would be interference with virus-maturation in analogy to the reported inhibition of Gag maturation by high levels of HIV-1 Vif [14]. Such a model seems particularly attractive as APO3G was found to interact with viral Gag precursor proteins [15].
